# Integrating Autoencoder and Heteroscedastic Noise Neural Networks for the Batch Process Soft-Sensor Design

**DOI:** 10.1021/acs.iecr.2c01789

**Published:** 2022-09-05

**Authors:** Sam Kay, Harry Kay, Max Mowbray, Amanda Lane, Cesar Mendoza, Philip Martin, Dongda Zhang

**Affiliations:** † Department of Chemical Engineering and Analytical Science, University of Manchester, Oxford Road, Manchester M1 3AL, U.K.; ‡ Unilever Research Port Sunlight, Quarry Road East, Bebington CH63 3JW, U.K.

## Abstract

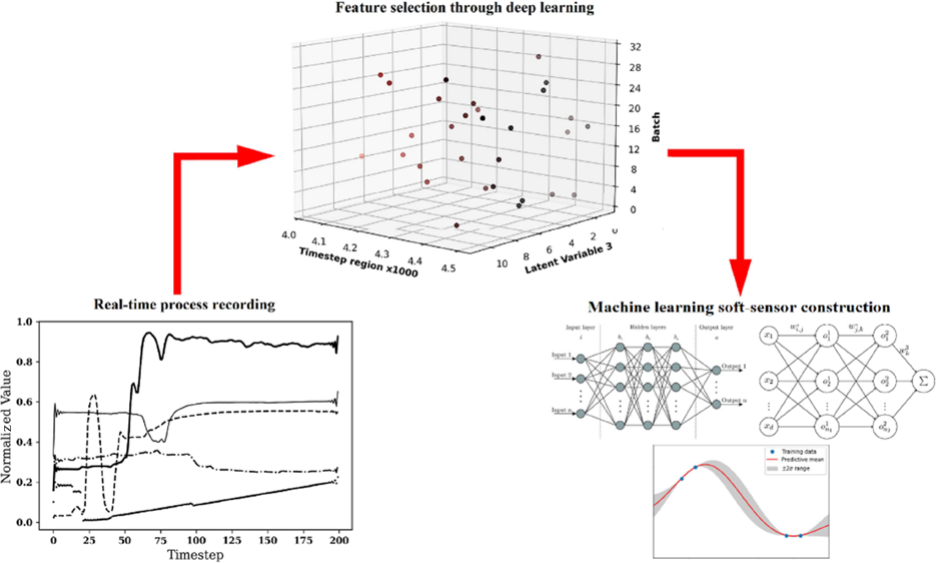

Viscosity represents a key product quality indicator but has been difficult to measure in-process in real-time. This is particularly true if the process involves complex mixing phenomena operated at dynamic conditions. To address this challenge, in this study, we developed an innovative soft sensor by integrating advanced artificial neural networks. The soft sensor first employs a deep learning autoencoder to extract information-rich process features by compressing high-dimensional industrial data and then adopts a heteroscedastic noise neural network to simultaneously predict product viscosity and associated uncertainty. To evaluate its performance, predictions of product viscosity were made for a number of industrial batches operated over different seasons. Furthermore, probabilistic machine learning techniques, including the Gaussian process and the Bayesian neural network, were selected to benchmark against the heteroscedastic noise neural network. Through comparison, it is found that the proposed soft-sensor has both high accuracy and high reliability, indicating its potential for process monitoring and quality control.

## 1 Introduction

The advancement and application of machine learning and data-driven models are major themes within the 4th Industrial Revolution. They are present in the development of many novel technologies, with substantial interest on the implementation of these techniques into process industries. The adoption of novel machine learning algorithms into manufacturing industries has yielded promising results and conveyed the benefits of utilizing such techniques in areas including scheduling, planning, and plantwide control.^1^ Increasingly, the process industries form large parts of the global economy. As ever, there is necessity for the development of efficient and reliable systems in which profit can be maximized to allow for companies to remain competitive within such environments. The development and uptake of machine learning algorithms promises to aid these developments.

In many chemical process industries, product analysis occurs directly by extracting samples (offline analysis) and using equipment to quantify a property indicative of product quality. This process can be slow and inaccurate depending on the conditions in which the sample and analysis are conducted, so clearly, an opportunity for improvement is available for product quality analysis. A soft sensor operated using real-time data would provide extensive benefits in such an environment by allowing predictions to be made in a fast and reliable manner.^2^ For instance, quality control is paramount to many formulated product industries to avoid the viscosity of the final product falling outside the predetermined acceptable boundaries; otherwise, the entire batch must be discarded, effectively wasting the entire process time. Not only does this lead to excessive losses due to material wastage but it also incurs costs involved with safe disposal of the defective products. Ideally, advanced in-line measuring equipment should be available to monitor the progress of the batch over the process time; however, these process analytical techniques have not been widely applied in industrial systems as they are expensive and difficult to be installed in an existing plant.^3^

As a result, building soft sensors becomes a perfect alternative solution. There are two general classes of soft sensors, one being physics (e.g., first-principle model)-driven and the other being data-driven.^4^ The physics-driven family has predominantly been applied to the design and planning of processing plants focusing on ideal steady-state operation. The data-driven soft-sensor overcomes this drawback as they are built using the data obtained during plant operation, which gives a better representation of the true process conditions, allowing them to be described in a more meaningful manner.^5^ This provided means for widespread adoption of data-driven soft sensors in batch operation systems with its most dominant application being the prediction of process key performance indicators.^3^

Specifically, real-time prediction of viscosity has historically been a challenge within the consumer goods industry.^6^ The difficulties stem from the current lack of understanding of rheology within the context of highly viscous fluids, making it impractical to derive any accurate physical models for viscosity prediction.^7^ It is common for industrial processes to take samples during a process to directly measure the viscosity. However, this is time consuming and if poor batch quality is observed, there is little opportunity to adjust the process to prevent deviations of viscosity outside acceptable boundaries. A solution to this can be applied with the usage of data-driven models that find the underlying relations that lie within the data recorded from a series of sensors on a plant and the measured viscosity. These data-driven models are then used as a soft-sensor for new process viscosity monitoring. In addition, to mitigate false confidence, these data-driven soft sensors should be able to make accurate estimations and represent the uncertainty present within the data. The uncertainty present within data is analogous to the uncertainty specified within the instrument used to record the viscosity values and the natural variation of the process itself.^8^

Therefore, in this work, we proposed a novel soft-sensor constructed by integrating state-of-the-art artificial neural networks to resolve the above challenges. Moreover, the generalizability of this soft sensor was further explored by using it to predict the viscosity of a different product variant. To better demonstrate the contribution of this work, the rest of this study is structured as follows. Section 2 describes the problem statement, Section 3 outlines the methodology, Section 4 presents the results alongside a thorough discussion, and finally, Section 5 concludes the current research finding.

## 2 Problem Statement

**Figure 1:**
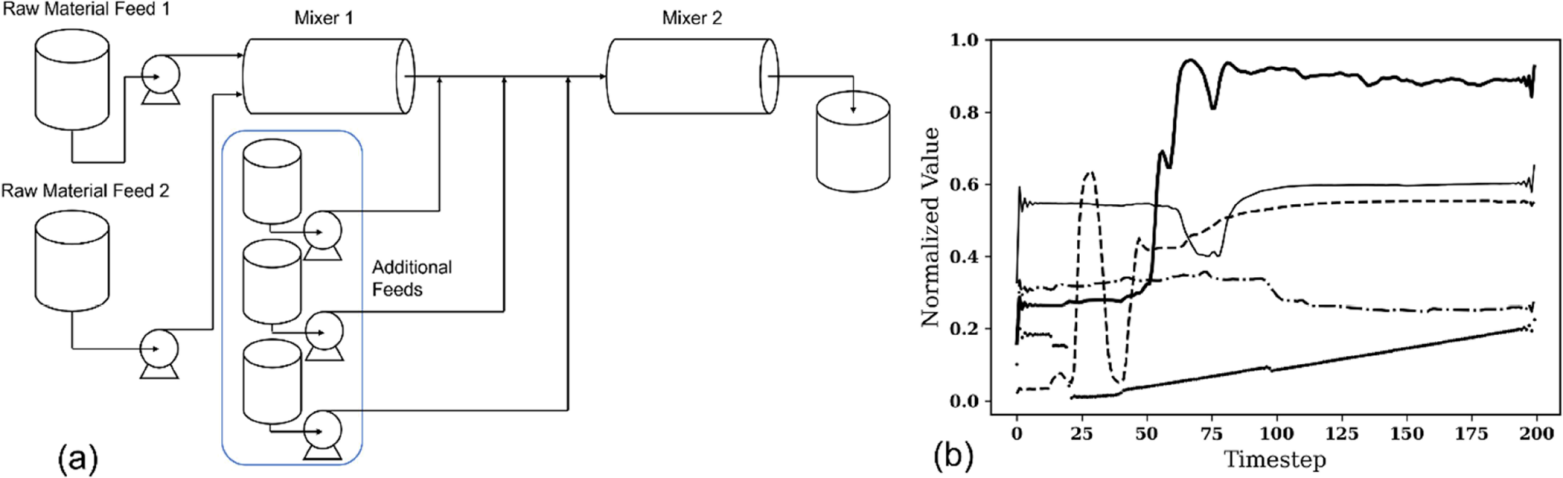
Diagram of the process under study (a) and example normalized sensor data for batch 1 of the α data set of the full process time (b).

This study focuses on a batch mixing process (shown in Figure 1a) for consumer goods product production.^9^ The aim is to develop a robust soft-sensor for final viscosity prediction using real-time process sensors’ recordings. Three data sets are collected from a manufacturing site and are referred to as the α, β, and γ data sets, respectively, where the first two were obtained from the same process line and the latter being obtained from a similar, but different process (i.e., producing a product variant). These data sets contain 30, 16, and 11 batches, respectively. Each batch contains 28 sensors recording temperatures, pressures, and flowrates at different locations in the process. The actual batch process generates around 7000 times series data points, and real-time data are recorded once per second (α data set) or once per two seconds (β and γ data sets). Figure 1b shows an example of the batch data.

In this work, the α data set was used for soft-sensor construction and cross-validation, the β data set was used for soft-sensor validation, and the γ data set was used to examine the soft-sensor’s generalizability. To build an effective soft-sensor, this work proposes an in-depth examination of the use of nonlinear dimensionality reduction techniques in conjunction with various machine learning regression models for viscosity prediction where latent variable modeling,^10^ advanced neural networks,^11^ and Gaussian processes^12,13^ are used. These techniques become especially useful when the relationships between the available process measurements and the output variable are best represented by nonlinear functions.

## 3 Methodology

### 3.1 Methodology Overview

Dimensionality reduction is necessary in this case to mitigate problems associated with high-dimensional data analysis^14^ and to improve accuracy of the data-driven soft sensor. Dimensionality reduction removes multicollinearity, improves the capability of the model to generalize to new data sets, and will eliminate redundant features whilst retaining important statistical relationships expressed within the data. Here, we offer the usage of an autoencoder for dimensionality reduction as opposed to traditional linear decomposition techniques such as principal component analysis (PCA) and partial least squares (PLS). Although the use of autoencoders is well demonstrated in a number of recent studies for data compression and outlier detection,^15^ for instance,^16^ proposed an autoencoder for complex multiscale heterogeneous material simulation, the implementation in batch process soft-sensing and monitoring has not been well explored. Once key process features are extracted through dimensionality reduction, we adopted either a heteroscedastic noise neural network (HNN), a Bayesian neural network (BNN), or a Gaussian process (GP) to make accurate viscosity predictions and meaningful uncertainty estimations. Successful predictions should have an error ∼10% to the industrial data considering the practical measurement errors within the factory. In addition, to effectively identify the best structure for the autoencoder, HNN, and BNN, Bayesian optimization is performed to optimize the hyperparameters of these models.

The primary reason to choose the three machine learning methods is to compare the validity of the assumptions behind the frequentist approach and probabilistic models. Heteroscedastic neural networks (i.e., the frequentist approach) assume a nonconstant variance in the residuals enabling the model to express data uncertainty, whereas both the probabilistic models (BNN and GP) naturally express uncertainty, both within the data and that which arises due to a lack of information (i.e., information uncertainty). With regard to the GP and BNN, we compare the technicalities of parametric versus nonparametric models with the BNN output being a function of its architecture.

### 3.2 Data Preparation

Regularly, within the recording of original data for batch process systems, redundancy exists. This usually manifests in the form of batch data sets (i.e., real-time measurements of process variables) that are not full rank. Therefore, it can be assumed that a reduced data set exists that is sufficiently representative of the inherent characteristics of the original data set. In our previous work,^9^ PLS was used to identify commonly important sensors and critical time regions that are preliminarily related to product viscosity within the data sets, leaving us with only information relevant to soft-sensor construction. This initial data analysis reduced all 3 data sets to a 3-rank tensor of *N* batches, *T* timesteps, and *J* sensors (*N* being dependent on the data set), where *T* and *J* are 300 and 8, respectively (reduced from 7000 timesteps and 28 sensors recorded in the original recordings). Before being used for training or validation purposes, the three-rank tensors are timewise unfolded to generate a matrix for soft-sensor construction.

It is possible to employ well established techniques that utilize linear transformations such as PLS and PCA when attempting to identify a latent space representative of the original data set. This, however, may not accurately portray and capture any nonlinear properties intrinsic to the process.^17^ Therefore, we propose the use of more novel nonlinear dimensionality reduction techniques, namely, the autoencoder. In an autoencoder, the subspace is identified without correlation to the batch product quality (i.e., the target we would like to predict, unlike in PLS); however, it is expected that the autoencoder has the potential to fully express any nonlinear relationships within the sensor data, thus reproducing a latent space more illustrative of the critical data selected through initial data screening.

### 3.3 Autoencoder Construction

**Figure 2:**
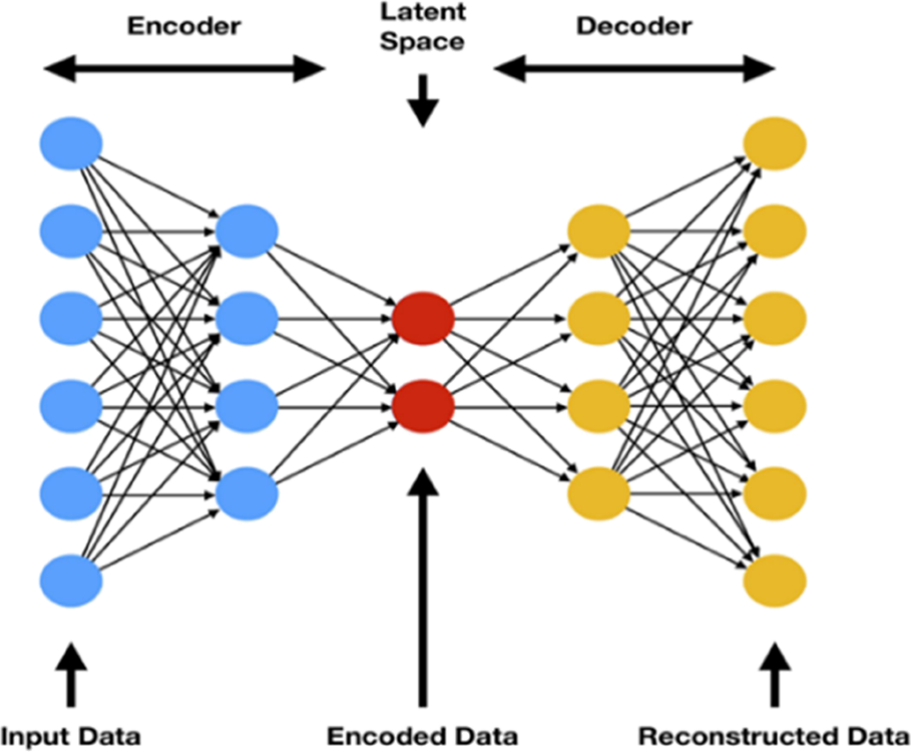
Diagram of a general autoencoder and its respective components.

First, we will introduce the structure of the autoencoder. For a given input (the process data) matrix ***X***, we obtain a projection ***Z*** and a reconstruction ***X***′. As illustrated in Figure 2, an autoencoder is defined by two neural networks:^18^ an encoder which is defined by a function *F*(***X***) = ***Z***, where ***X*** and ***Z*** are the respective inputs and outputs of the network and a decoder which is defined by a function *G*(***Z***) = ***X***′, where ***Z*** and ***X***′ are the respective inputs and outputs of the network. The training objective of an autoencoder is defined by a cost function in which the distance between the input and its reconstruction error is to be minimized. Henceforth, the cost function assumed for the construction of the autoencoder will be the mean squared error; for an input of *n* datapoints, the error  and the activation function applied to all hidden layers is ELU (exponential linear unit) defined by .^19^ A condition must be enforced on the model to copy the input data as its output (i.e., reconstruct the data set), in the interest of extracting useful properties and characteristics from the process data.

The structure of an autoencoder is of critical importance to its performance. It was clear that a maximum and minimum limit must be imposed on the acceptable number of latent variables that can be used within machine learning models to predict batch quality. If these bounds are not set, significant problems arise with trivializing the data by eliminating correlations and causing difficulties with interpretation within the predictive models. To identify the optimal autoencoder structure, Bayesian optimization was employed to optimize the number of layers and neurons within each layer as well as other hyperparameters including the learning rate and the number of epochs. A detailed introduction to Bayesian optimization can be found in a previous study.^20^ It was found that there are two specific structures resulting in the same minimum loss function; henceforth, they were both adopted in this work for further investigation and summarized in Table 1 and named Autoencoder 1 and Autoencoder 2, respectively. These two autoencoders represent 16 and 4 latent variables, respectively. The methodology for constructing the autoencoder employed in this study does not require prior knowledge of any previous data sets, meaning that the model structure and hyperparameters were adaptable to the specific data set from which the latent space was being extracted.

**Table 1:** Representation of the Parameters Used for the Construction of the Autoencoders

	hidden layer	number of nodes	learning rate	epochs
autoencoder 1:16 latent variables	1	1404	1.477 × 10^–3^	782
	2	94		
	3	50		
	4	16		
	5	50		
	6	94		
	7	1404		
autoencoder 2:4 latent variables	1	977	2.836 × 10^–3^	884
	2	231		
	3	48		
	4	4		
	5	48		
	6	231		
	7	977		

### 3.4 Data-Driven Regression Model Construction

#### 3.4.1 Heteroscedastic Noise Neural Network

Let us now describe the details of how the HNN generates viscosity predictions. HNNs employ the traditional approach to predictive applications using artificial neural networks but differ in that they use probabilistic modeling to represent the data uncertainty, which results from the random nature of the studied system, and is irreducible.^21^ The ability of HNNs to provide a meaningful uncertainty estimate for each prediction is attributed to their modified loss function (eq 1) in which a Gaussian negative log likelihood term is embedded within the traditional ANN’s loss function:^22^
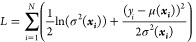
1where σ(***x*_*i*_**) represents the estimated standard deviation of the HNN for input (latent vector) ***x*_*i*_**; *y_i_* is the measured value of viscosity for ***x*_*i*_**; and μ(***x*_*i*_**) is the predicted viscosity for ***x*_*i*_**.

#### 3.4.2 Bayesian Neural Network

BNNs are another variant of ANNs built upon the belief that the frequentist approach to ANN predictions does not fully characterize the data set (i.e., the model cannot be represented by a single structure with fixed parameters), so it is necessary to represent the model parameters with a probability distribution.^23^ The distributions are applied to model parameters via Bayesian inference, where the prior beliefs about the parameters are updated to posterior distributions after new information becomes available. For example, *D* corresponds to the newly available data and *p*(θ) to the prior beliefs about the parameters, whose probabilities may be updated upon receiving new data. This is expressed via Bayes’ theorem:
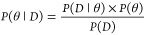
2where *P*(θ) represents the probability that the prior beliefs hold true, *P*(*D* | θ) is the probability of observing new data given the prior beliefs and holds within the data uncertainty of the model, *P*(*D*) is the probability that new data will be observed, and *P*(θ | *D*) is the probability that the prior beliefs hold, given the newly available data.^24^ In practice, a BNN is trained approximately. One approach is encompassed by the evidence lower bound (ELBO). The idea of the ELBO is to learn an approximation of the true posterior. Maximization of the ELBO, which acts as an objective function for BNN training, minimizes the distance (specifically, the KL divergence) between the true posterior and the approximation that we seek to identify. Typically, the approximating posterior is chosen to belong to the family of Gaussians such that once identified the posterior distribution over parameters is parameterized by a mean and variance. This is different to the conventional ANN, which assumes a point estimate for each model parameter. A detailed introduction to BNN construction can be found in a previous study.^25^

#### 3.4.3 Gaussian Process

A GP is a type of continuous stochastic process defined as a set of random variables, represented by matrix ***F***, with inputs represented by matrix ***X*** such that all finite subsets of the random variables follow a multivariate normal distribution.^26^ A GP is considered to be nonparametric, where the parameters of the model are set by the input data set, from which it maps nonlinear features from the input to the output matrices.^27^ Similar to the BNN, the GP is fully probabilistic and relies on Bayesian inference for its predictive ability. A difference between the BNN and the GP’s application of Bayesian inference is that a GP applies a prior over the input–output relationships as opposed to the BNN’s application prior to unknown parameters. A prior in a GP is specified through a mean and a covariance function where the covariance function relates the covariance between random variable pairs.^26^ In this work, the GP covariance function was selected as a Matern 5/2 kernel, with the associated parameters estimated by maximizing the marginal log likelihood.^28^ A detailed introduction to GP construction can be found in a previous study.^29^

#### 3.4.4 Metrics for Comparison

It is essential that appropriate metrics are chosen to evaluate the probabilistic predictive models. This allows for a consistent, transparent comparison between each model. The objective is to accurately predict batch quality and associate each prediction with an uncertainty metric representing the confidence of the model in its predictions. Hereafter, the following metrics will be defined to establish a consistent method of comparison between soft sensor models.

The accuracy of the predictive models will be estimated using the mean average percentage error (MAPE):
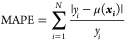
3

The distribution of potential viscosity values is measured as the percentage uncertainty, which can also be interpreted as a scaled coefficient of variation:
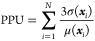
4

It is important to note that the PPU was not considered to be a priority in determining the best performing model as long it is approximately 25–40%. This range represents the variation of the data generating process, which has been estimated from offline viscosity measurements within the factory.

Intuitively, the model’s uncertainty estimate should also cover the deviation from the mean prediction to the industrially measured viscosities with a given probability. The probability with which a model achieves this is known as the coverage probability. The overall probability that a model’s prediction will lie within a region close to the true viscosity measurement is known as the coverage probability.^30^ This region is defined by the model’s uncertainty boundaries, and the coverage probability is expressed as:
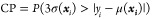
5

Given that the predictive distributions constructed by the soft sensor are (approximated) as Gaussian, and both the CP and PPU are defined with 3σ(***x***_*i*_), in theory, CP > 0.997.^30^ However, because of the limitations of samples and potential for the true data generating distributions to be unsatisfactorily described by a Gaussian, a credible predictive model was deemed to satisfy CP > 0.8.

#### 3.4.5 Model Training and Validation

The HNN and the BNN utilized the same Bayesian optimization scheme as the autoencoder in order to identify their best structure and hyperparameters.^31^ All the predictive models were trained using the α data set, which consists of 30 batches. The model structure performance was evaluated using cross-validation techniques, namely, “leave 2 out” cross-validation.^32^ This involved isolating a subset of two unique batches from the original set for validation and forming a new data set containing the other 28 batches, which was used for training. Overall, there are 435 combinations of training and validation subset splits to train on and predict, so the average performance is calculated by averaging over the different combinations. This method increases the accuracy in estimating the general predictive capabilities of a given model structure because it minimizes bias from evaluating on a single training and validation split. Once cross-validation of the soft sensors was completed and the model structure was selected, the β and γ data sets were used to validate and compare performance of all the three soft sensors.

It is worth noting that when the soft sensor is applied to monitor a process with a different product or a different sampling time, it is possible that the soft-sensor’s performance is deteriorated. However, in this study, based on the industrial experience, it is confirmed that data sets β and γ are sufficiently similar to data set *α* such that the quantitative metrics are the same (MAPE ≤ 15%, 20 ≤ PPU ≤ 30, CP ≥ 0.8). It should be also noted that unlike the constraints on the CP and MAPE, the constraint on the PPU was not definitive and was intended to act as a guide, so small deviations were acceptable.

In this work, the autoencoder and HNN models were implemented using PyTorch v1.10.0; the GP models used GPy v1.9.9, and the BNN models were constructed using torchbnn v1.2.0. Mathematical operations and data formatting were completed using NumPy v1.20.3, Pandas v1.3.4, and Matplotlib v3.4.3.

## 4 Results and Discussion

The final model structures of the HNN and BNN after Bayesian optimization were extracted and summarized in Table 2. The cross-validation performance of the three soft sensors is also listed in Table 2. It is noted that for very few sets using 16 latent variables, the GP does not perform well. This will be discussed in detail in Section 4.2.1.

**Table 2:** Structures and Performance of the Soft Sensors alongside the Cross-Validation Results on the α Data set^a^

regression model	HNN	BNN	GP
number of hidden layers	2	2	N/A
number of nodes [layer 1, layer 2]	[31, 3]	[2 J, 2]	
learning rate	0.0125	0.01	
activation function	Sigmoid	ReLU	
number of epochs	160	100 + 50 J	
MAPE % [training, validation] – 16 LV	[7.8, 10.0]	[9.4, 12.1]	[9.9, 9.3]
PPU % [training, validation] – 16 LV	[28.9, 28.3]	[0, 5.53]	[23.1, 22.9]
CP [training, validation] – 16 LV	[1, 1]	[0, 0.22]	[1, 0.94]
MAPE % [training, validation] – 4 LV	[10.2, 12.0]	[10.7, 11.6]	[11.1, 9.7]
PPU % [training, validation] – 4 LV	[36.3, 36.4]	[0, 2.8]	[25.3, 25.3]
CP [training, validation] – 4 LV	[1, 1]	[0, 0.16]	[1, 0.99]

a *J* refers to the number of latent variables. Results are dependent on the use of the specific autoencoder (one with 16 latent variables, the other with 4 latent variables).

### 4.1 Results of the HNN-Based Soft-Sensor

The final structure of the HNN used for validation was identical for both the 16 and 4 latent variable autoencoders with the exception of the size of the input layer. The HNN structure, activation function, epochs, MAPE, PPU, and CP are shown in Table 2.

#### 4.1.1 Validation Using 16 Latent Variables

**Figure 3:**
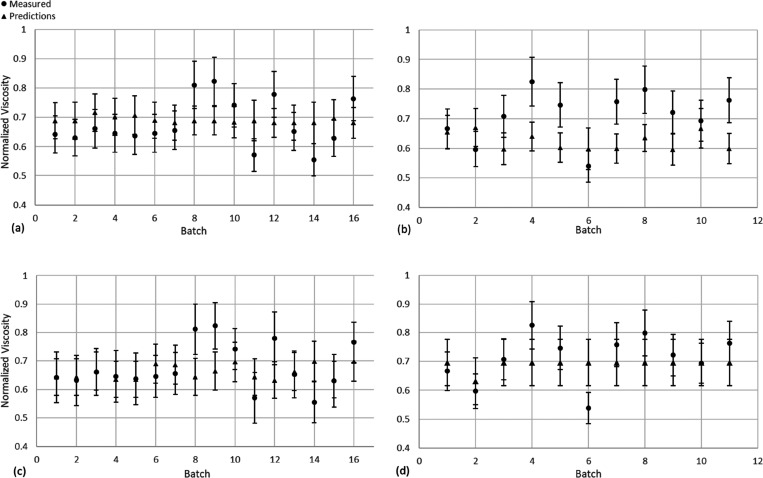
HNN soft sensor predictions against the measured values for data sets β (a) and γ (b) with 16 latent variables, and for data sets β (c) and γ (d) for 4 latent variables. Error bars represent one standard deviation.

The predictions of the soft sensor have been plotted in Figure 3 with error bars representing one standard deviation. As shown in Figure 3a, the soft sensor is capable of predicting β data set’s batch quality to a high degree of accuracy on data derived from the same process as the training set (α), with an average MAPE of 11.3%. Similarly, the uncertainty estimations average to ±26.0% for three standard deviations (±8.67% for one standard deviation). Notably, 88% of the datapoints for the measured viscosities are contained within 2 standard devitaions of the predictions. This means that the soft sensor’s predictions are lying within the acceptable range of values determined from the experimental procedure; the expected standard deviation of measurements taken for the viscosity is >±8% because of the standard experimental errors (i.e., uncertainties of measurement equipment and human error in experiments). The significance of this is such that the soft sensor has successfully replicated the error within the process data in its predictions without overfitting the measurement noise. The MAPE, PPU, and CP for the HNN’s validation results of the β data set can be found in Table 3.

**Table 3:** Results of the HNN-Based Soft-Sensor When Validating on β and γ

	data set	MAPE %	PPU %	CP
16 latent variables	β	11.3	26.0	1
	γ	15.5	26.8	1
4 latent variables	β	8.3	35.1	1
	γ	10.1	30.4	1

A similar conclusion can be drawn of the model capacity to predict the batch quality of the γ data set (different product variant, see Figure 3b) as to that of the β data set. The results indicate slightly worse performance on γ with an average MAPE of 15.5%; this, however is expected due to the data being derived from a different process. The model also seems to provide less overlap between measured data points and the uncertainty associated with the predictions, with only 55% of them overlapping for two standard deviations. However, this value is increased to 100% for three standard deviations, indicating that the soft sensor is still able to provide a reasonable degree of reliability. The MAPE, PPU, and CP for the HNN’s validation results of the γ data set can be found in Table 3.

#### 4.1.2 Validation Using Four Latent Variables

The predictions of the soft sensor have been plotted on Figure 3c, d with error bars representing one standard deviation. From Figure 3, it is clear that the four latent variable HNN model possesses satisfactory predictive capabilities with averaged MAPE values of 8.3% and 10.1% pertaining to the β and γ data sets, respectively. The model predictions and the measured values have significant overlap for almost every batch using one standard deviation (displayed as error bars). These results suggest a high degree of accuracy for the model’s predictions, which provide confidence in the HNN’s proficiency in predicting batch quality.

However, it is noted that there is little difference in the HNN’s predictions across the different batches in the γ data set. There are several possible explanations for this behavior, one of which is that this reflects a lack of information being captured in the dimensionality reduction process; as can be seen in later sections of the paper, this is a recurring issue discussed in length. In this analysis, it is evident that the 4 LV HNN is a promising contender for its use in early estimation of batch quality for industrial processing. A quick comparison with the 16 LV HNN results reveals a decrease in MAPE and an increase in PPU; this is to be expected as using less information to train the HNN will lower the risk of overfitting to the training data set (corresponding to the lower MAPE), but will also lower the confidence of its prediction (corresponding to the higher PPU); hence, it will increase the model’s accuracy but reduce its confidence in its application to new data sets, provided the nature of the new data set shares similarities with the training data set. However, as MAPE is a more important metric than PPU, the current result suggests that the 4 LV-based HNN soft-sensor is more promising for future industrial applications.

Overall, it was anticipated that the HNN would provide better performance on the α and β data sets than on the γ data because of them both being obtained from the same process line, meaning their characteristics should be similar. Even though the data sets were obtained at different times of the year with different recording frequencies (α data set recorded once per second, β data set recorded once per two seconds), the performance on the β data set would indicate that this had little effect on the model’s capacity to accurately predict batch quality, thus meaning that the inherent features of the process data are similar. In Figure 3a, d, it can be seen that there is little variety in the estimation of viscosity made by the HNN soft-sensor. This could be attributed to the nature of the autoencoder as viscosity is not taken into account when extracting the feature space of the data set provided. This leaves the possibility that the important physical relations between the sensor data and the viscosity, that are necessary for making accurate predictions, are partially lost when reducing the dimensionality. A second explanation could be that there is no identifiable difference between the features of each batch that would give rise to a reason for the model to predict largely different viscosities, as ideally all the batches should be operated under similar conditions so that product quality should be close to the specification target.

### 4.2 Results of the GP-Based Soft Sensor

#### 4.2.1 Validation Using 16 Latent Variables

For GP training, Batch 26 presented itself as an outlier, and thus, the MAPE and PPU were calculated both with and without its inclusion. Including Batch 26, the MAPE and PPU for the α data set were 147.8% and 185.8%, respectively. Excluding the outlier, the values become much closer to the expected results being 9.25% and 22.92%. The coverage probability was in the acceptable range with its value being 0.94. It is worth noticing that Batch 26 was not found to be an outlier when building the HNN soft-sensor, suggesting that the GP soft sensor could be more sensitive to changes of data and may be valid within a narrower operational range.

Unfortunately, the model failed to generalize with respect to accurately predicting the viscosity of the β and γ batches with the mean absolute percentage error of β being close to 29,000% and γ being close to 1600%. The coverage probability for both scenarios was 0. Upon further investigation into why this occurred, it was observed that there was a large degree of separation between the data sets identified in terms of the latent space. This could have caused the model to incorrectly identify and misinterpret correlations, resulting in large inaccuracies in the predictions of the batch viscosities.

#### 4.2.2 Validation Using Four Latent Variables

**Figure 4:**
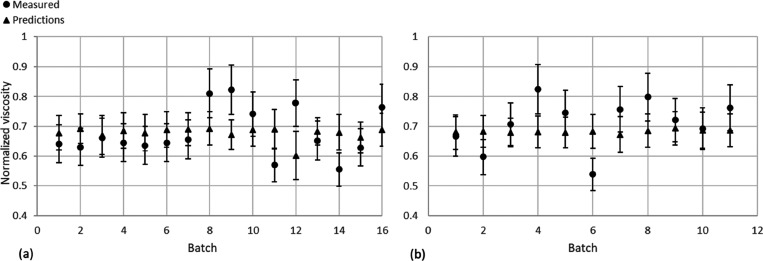
GP soft-sensor predictions against the measured values for batch data for data sets β (a) and γ (b) using a GP with four latent variables. The error bars represent one standard deviation.

The predictions of the GP soft sensor have been plotted in Figure 4 with error bars representing one standard deviation. The results are as shown below in Figure 4a, b and are tabulated in Table 4.

**Table 4:** Results of the GP-Based Soft Sensor When Validating on β and γ

	data set	MAPE	PPU	CP
4 latent variables	β	10.5	26.0	0.94
	γ	10.3	23.8	1.0

Unlike when using 16 latent variables, the use of smaller latent spaces drastically improved the performance of the GP soft sensor, making it a competitive option. The predictive capabilities of the GP are satisfactory with MAPE and PPU values of 10.5% and 26.0% for the β data set and 10.3% and 23.8% for the γ data set. These uncertainty estimates are fairly representative of the data uncertainty contained within the data set, which has been evaluated to be approximately 30% (three measurement standard deviations). In accordance with the comparison metrics, a model can be defined to be suitable if CP > 0.8. This is fulfilled by the GP, possessing high coverage probabilities of 0.94 when validating on the β data set and a value of 1 when validating on the γ data set. In Figure 4b, it was observed that predictions on γ maintained a relatively constant value with little deviation from a set point, whereas β predictions exhibited increased variation. This phenomenon was observed within the other machine learning models and is discussed in Section 4.4. Overall, this outcome provides confidence in the GP soft sensor, presenting high accuracy and high coverage probabilities while generalizing well to data sets obtained during different seasons and process lines.

In addition, a further investigation reveals that the MSE (i.e., mean squared error) in the reconstruction of the autoencoder is similar for the 16 and 4 LV; therefore, it can be interpreted that the decompositions capture similar amounts of information. This is reinforced by the sparsity in the 16 LV latent space, indicating that a degree of redundancy exists when compared to the 4 LV latent space, which was found to be more densely populated. It is known that GPs perform well with low dimensional input spaces because of the nonconvexities associated with GP training, so it is intuitive that the GP will perform worse using the larger latent space.

### 4.3 Results of the BNN-Based Soft-Sensor

The BNN structure, activation function, epochs, MAPE, PPU, and CP are shown in Table 2, where the testing results indicate poor uncertainty coverage. Although the percentage errors seem acceptable, the lack of capability for the model to estimate the inherent error associated with its predictions leads to a lack of reliability in its application as a soft sensor.

#### 4.3.1 Validation Using 16 Latent Variables

**Figure 5:**
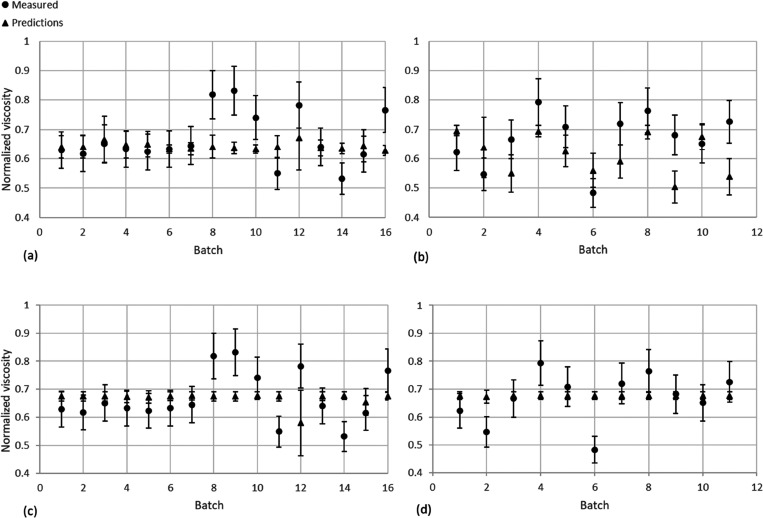
Plots of the soft sensor predictions against the measured values for batch data for data sets β (a) and γ (b) using the BNN with 16 latent variables and for data sets β (c) and γ (d) with 4 latent variables. The error bars represent one standard deviation.

The predictions of the BNN soft sensor have been plotted in Figure 5 with error bars representing one standard deviation. From Figure 5, it can be reasoned that the BNN-based soft sensor fails to serve its purpose as, for predictions on both the β and γ data sets, the coverage is unacceptably low with values of 0.56 and 0.18, respectively. This result is significant in that there is an unreasonable overconfidence in the soft sensor’s predictions, which fails to represent the hypothesized probability distribution of viscosity values. Despite this, the MAPE of the β data set is comparable to that of the HNN boasting a value of 10.3%; however, as can be seen in Figure 5a, there is little variation in the value of the predictions generated by the BNN model as it has seemingly chosen a mean viscosity value from the inputted training data as an attempt to increase the accuracy of the soft sensor. When the γ data set is used for validation, the responsiveness of the model output to model input is increased over data set β. This is shown by Figure 5b; a potential reason for this occurrence is that the γ data are obtained from a different product than the training data so there may be noticeable variability between the input data provided to the BNN.

#### 4.3.2 Validation Using Four Latent Variables

The predictions of the soft-sensor have been plotted in Figure 5 with error bars representing one standard deviation, and the important metrics are shown in Table 5.

**Table 5:** Results of the BNN-Based Soft-Sensor When Validating on β and γ

	data set	MAPE	PPU	CP
16 latent variables	β	10.3	6.0	0.56
	γ	17.6	8.6	0.18
4 latent variables	β	12.3	3.9	0.06
	γ	10.0	2.1	0.18

Similar to the conclusion of the BNN-based soft sensor model developed using 16 latent variables, constructing the model using 4 latent variables results in a failure to serve its purpose. The BNN consistently generated low uncertainty estimations giving rise to low coverage probabilities as defined previously in Section 3.3.1 thus, it follows that little reliability can be held in the model’s predictions of batch quality. In addition, the model suffers from an identical problem to the BNN trained using 16 latent variables where the selection of a mean viscosity value would be prioritized when reducing error rather than attempting to replicate the true trend. A possible reason for the model’s failure to replicate the viscosity trend is that there is too little information available within the latent variables provided. Evidence to support this is found in Figure 5b, where the soft sensor predictions (constructed using 16 latent variables) vary significantly. Therefore, it may be true that for a BNN, larger latent spaces are required to develop a more accurate soft sensor.

### 4.4 Soft Sensor Comparison and Selection

From the results shown in Section 4.3, it is clear that the BNN-based soft sensor fails regarding its functionality for batch quality prediction because of its poor representation of the inherent uncertainties present within the data sets. Therefore, the selection process is reduced to a comparison of the HNN-based soft-sensor and the GP-based soft-sensor and between using 4 and 16 latent variables. Given that the GP-based soft-sensor is unable to function accordingly when using the 16 LV derived from the autoencoder, only the 4 LV model will be considered for this application. It is noticed that for some data sets the HNN-based soft-sensor yields nonvarying batch quality predictions over all the batches, as shown in Figure 3d. A similar behavior of predicting constant viscosity was also observed for the BNN- and GP-based models, so it is not possible to disregard the importance of this phenomena, and any physical significance must be investigated. The uncertainty associated with the industrial viscosity measurements is moderately high, and it is plausible that the model is unable to determine any statistically significant difference between viscosity of each batch for a given data set, which would give rise to a constant (i.e., averaged) viscosity prediction. Notably, when validating on the γ data set using four latent variables, all three machine learning models’ predictions were almost invariant; to determine the cause of this, the extracted latent space for this data set was studied, revealing almost constant values across all the batches. Therefore, it is possible that the true viscosity of this data set is the same over all batches, but due to measurement error, they were recorded as different values. Within the scope of this study, preference is given to varying batch quality predictions because it cannot be confirmed that the explanation of a universally constant viscosity is true.

It is noted that the uncertainty estimates for each of the models under study lies within the 30 to 40% region for three standard deviations. This is acceptable because it reflects the inherent uncertainty in the obtained industrial data, which is estimated to be between ±8 and ±13.3% for one standard deviation (see Section 3.4.4). Therefore, there is no apparent difference between the different model capabilities in estimating the uncertainty associated with a prediction, and so this metric is neglected from further analysis in this comparison. The coverage probability for all GP- and HNN-based models is close to 100% in all cases; this, alongside the reasonably low MAPEs, provides confidence that all models are suitable for the desired application, so the selection of the most appropriate soft sensor becomes strictly a case of which model provides the lowest percentage errors with varying viscosity predictions for the γ and β data sets.

Both the GP (4 LV) and HNN (16 LV and 4 LV) soft sensors display satisfactory predictive capabilities. When predicting the β and γ data sets, the GP has a MAPE of 10.5% and 10.3%, respectively. The HNN models (16 LV and 4 LV) possess MAPEs of 11.3% and 8.3% for the β data set, respectively, and 15.5% and 10.1% for the γ data set. Although, very useful in determining the effectiveness of a model, the MAPE does not convey outliers within the data set. Ideally, the chosen model should be consistent in its predictions over all batches (accounting for expected variation between batches), and so, the results from each model are inspected for such outliers. It was determined that no model gave unreasonable predictions for a batch in any data set; the highest individual batch MAPE values are 23%, 27%, and 27% for the 16 LV HNN, 4 LV HNN, and the GP model, respectively. Thus, it is concluded that no model provides any outliers significant enough to warrant concern in the model’s consistency.

Using the average MAPE results, the 16 LV HNN-based soft-sensor provides the worst results; however, given that all three considered models provide similar MAPE’s, any further conclusion based on these small differences would be specific to this case study and so will not be made. The GP model provides almost identical MAPEs for both the γ and β data sets, even though the process from which γ is obtained differs from both α and β. This implies that the GP model is well generalized, thus removing the restriction of its use in processes similar to that of the training data set (α). The 4 LV HNN model gives slightly better MAPE results for the β predictions but slightly worse predictions for the γ data set, indicating that it is slightly less generalized; however, as this increase in MAPE is small, it is not reasonable to regard this as conclusive evidence outside of this case study. Therefore, through the current comparison, it was found that the 16 LV HNN soft sensor, the 4 LV HNN soft sensor, and the 4 LV GP soft sensor can provide good prediction accuracy and reliability for viscosity prediction. However, specific to the comparison of the GP and HNN, the HNN’s predictive ability is found to be more robust with respect to the changing of latent space dimensions, whereas the GP is only applicable to a low latent space dimension. As previously discussed, a lower latent space dimension may contain less information of the original process. Thus, using a soft senor which is applicable for higher latent space dimensions may offer greater flexibility and provide additional benefits if more data sets are available. As a result, the HNN could be considered as a better option in such cases.

Finally, to highlight the performance of the current soft-sensor, a soft-sensor designed in our previous work was used as a benchmark.^33^ In our previous work, MPLS was used as a linear dimensionality reduction technique feeding into either an HNN, BNN, or GP. The MPLS-GP results are used to benchmark with the results obtained from this autoencoder study as this soft sensor model performed the best with regard to the quantitative metrics. Comparing the MPLS-GP results with those of the 4 LV autoencoder-based HNN model, it can be found that the MPLS model provides a lower coverage of residuals because of increased confidence in its predictions (12% and 9% lower CPs for β and γ). Also, the MAPE of the autoencoder model is decreased by 1.7% and 1.3% for β and γ, respectively. Using the quantitative metrics to compare the two dimensionality reduction methods appears to suggest a slight improvement favoring the autoencoder; however, it has been observed that the MPLS-GP suffers from stoic predictions, more so than the autoencoder models. The MPLS-GP requires a smaller latent space to achieve its optimal predictions, so the lack of responsiveness could indicate loss of information when reducing the dimensionality of the input data so drastically. In contrast, the autoencoder study presents the HNN’s ability to use both larger and smaller spaces, increasing the model’s robustness.

## 5 Conclusions

In conclusion, autoencoders are a viable dimensionality reduction method and can be used in conjunction with machine learning regression models to build a robust soft sensor able to predict the outcomes of industrial processes such as batch quality. In our previous work,^33^ a PLS-based dimensionality reduction model was investigated but the robustness of the model was limited, presenting flaws in the sensor’s responsiveness and generalization capabilities. This is mainly because the investigated product is a highly viscous, non-Newtonian fluid; thus, using an autoencoder proved useful because the viscosity and the process variables are nonlinearly related. As shown in this work, the autoencoder can effectively produce small latent spaces, which accurately represent large data sets, thus removing the problems associated with high dimensionality model regression. Through the use of data-driven models and nonlinear dimensionality reduction techniques, it is possible to reduce the capital cost in expensive process analytical equipment, whilst identifying a high-quality solution to industrial problems. Through cross-validation, it is seen that the HNN and GP models have both high accuracy and high reliability when predicting viscosity.

The developed soft sensors were also found to be able to predict different processes operated over a broad time span with a relatively large viscosity variation, thus presenting a significant benefit over conventional linear regression-based dimensionality reduction and modeling methods. From the proposed machine learning models (HNN, BNN, and GP), it was concluded that the BNN was inadequate in its implementation as a soft sensor, whereas the HNN and GP succeeded in meeting all requirements defined by the metrics proposed in this work. Specifically, both models had high probability coverages, low errors, and uncertainty estimates that capture the variance in data generation. Meanwhile, the HNN offers extra flexibility when the number of latent variables cannot be firmly determined. Overall, this work demonstrates the innovative combination and potential impact of advanced artificial neural networks on industrial data analysis and batch process monitoring.

Finally, in terms of the practical use of the soft sensor, the soft sensor was trained and validated using the critical region and sensors identified. The critical time region is located shortly after the premixing phase of the batch (close to the beginning), and through latent variables analysis, it is found that there exists distinct difference between the critical region and the noncritical region. As a result, by monitoring the change of latent variables in real-time, it is feasible to activate the soft sensor in real-time for early batch quality predictions. Moreover, a statistics-based activator can be designed to systematically identify when to switch on and off the soft sensor. This will be investigated in future work.
